# Predictive Value of the Texture Analysis of Enhanced Computed Tomographic Images for Preoperative Pancreatic Carcinoma Differentiation

**DOI:** 10.3389/fbioe.2020.00719

**Published:** 2020-06-30

**Authors:** Zhang Longlong, Li Xinxiang, Ge Yaqiong, Wei Wei

**Affiliations:** ^1^Department of Radiology, Anhui Provincial Hospital Affiliated to Anhui Medical University, Hefei, China; ^2^Department of Radiology, The First Affiliated Hospital of USTC, Division of Life Sciences and Medicine, University of Science and Technology of China, Hefei, China; ^3^GE Healthcare China, Shanghai, China

**Keywords:** pancreatic carcinoma, texture analysis, contrast-enhanced CT, pathological grading, machine learning

## Abstract

**Purpose:**

To assess the utility of texture analysis for predicting the pathological degree of differentiation of pancreatic carcinoma (PC).

**Methods:**

Eighty-three patients with PC who went through postoperative pathology diagnose and CT examination were selected at Anhui Provincial Hospital. Among them, 34 cases were moderately differentiated, 13 cases were poorly differentiated, and 36 cases were moderately poorly differentiated. The images in the arterial and venous phase (VP) with the lesions at their largest cross section were selected to manually outline the region of interest (ROI) to delineate lesions using open-source software. A total of 396 features were extracted from the ROI using AK software. Spearman correlation analysis and random forest selection by filter (rfSBF) in the caret package of R studio were used to select the discriminating features. The receiver operating characteristic ROC analysis was used to evaluate their discriminative performance.

**Results:**

Twelve and six features were selected in the arterial and VPs, respectively. The areas under the ROC curve (AUC) in the arterial phase (AP) for diagnosing poorly differentiated, moderately differentiated and moderate-poorly differentiated cases were 0.80, 1, and 0.80 in the training group and 0.77, 1, and 0.77 in the test group; in the VP, the values were 0.81, 1, and 0.82 in the training group and 0.74, 1, and 0.74 in the test group.

**Conclusion:**

Texture analysis based on contrast-enhanced CT images can be used as an adjunct for the preoperative assessment of the pathological degrees of differentiation of PC.

## Introduction

Although all the efforts have been made to develop better pancreatic carcinoma (PC) treatment strategies, the prognosis still remains poor. PC, which is a tumor with a very high degree of malignancy and it is usually found at an advanced stage (Luu et al., 2019). Early diagnosis remains challenging if the tumor is not located close to the common bile duct, causing obstructive jaundice (Siegel et al., 2016). Some patients have already missed the best treatment opportunity when PC is discovered. PC cells are highly invasive and prone to metastasis and the 5-year survival rate is only 5–7% (Schima et al., 2007; Siegel et al., 2016). Wang et al. (2003, 2007) reported that the degree of enhancement and differentiation of the tumor were inversely proportional to the degree of malignancy. The lower the degree of enhancement, the higher the degree of malignancy, and the lower the degree of differentiation, the higher the degree of malignancy. Surgery is the only effective method to cure PC and is still considered for most lesions (Cloyd and Poultsides, 2015; Wong et al., 2018). Different pathological grades of PC have different prognoses (Kurihara et al., 2015; Matsumoto et al., 2015). Therefore, the preoperative prediction of the pathological grade and differentiation of lesions is very important in the treatment and prognostic evaluation of patients with PC (Kurihara et al., 2015; Matsumoto et al., 2015). PC can be detected with computed tomography (CT), magnetic resonance imaging (MRI), and ultrasound (US), but CT is still the most commonly used method for the diagnosis of PC (Chun-Ye et al., 2012). Recently, texture analysis technology has become a topic of growing interest. This technology is widely used in the diagnosis, differential diagnosis and pathological grading of diseases by extracting potential information from the image and analyzing the extracted texture features. When the radiologists diagnose the disease, it is mainly based on the observation of the image of the lesion and the clinical manifestation of the patient. The application of texture analysis in imaging can quantify the imaging features of the disease, thus assisting the imaging physician in the diagnosis (Davnall et al., 2012; Ng et al., 2013; Rao et al., 2014; Hanania et al., 2016; Choi et al., 2018).

Some researches had shown CT texture analysis was helpful to the prediction of the resectability and prognosis in patients after neoadjuvant therapy for pancreatic ductal adenocarcinoma and the prediction of pancreatic neuroendocrine tumor grade (Canellas et al., 2018; Choi et al., 2018; Wong et al., 2018). Sandrasegaran et al. (2019) has reported that CT texture analysis was easy to perform on contrast-enhanced CT and it could determine prognosis in patients with unresectable PC. Huang et al.’s (2019) study showed that two-dimensional texture analysis was a feasible quantitative technique for the differential diagnosis of pancreatic lymphoma from pancreatic adenocarcinoma, and the diagnostic performance was similar to CT characteristics. To the best of our knowledge, texture analysis has not been used as a method for predicting PC differentiation. The purpose of this study is to explore the value of texture analysis on CT images for predicting PC differentiation.

## Materials and Methods

### Ethical Approval

The studies involving human participants were reviewed and approved by Medical Research Ethics Committee of The First Affiliated Hospital of University of Science and Technology of China (Anhui Provincial Hospital).

### Patients

**FIGURE 1:**
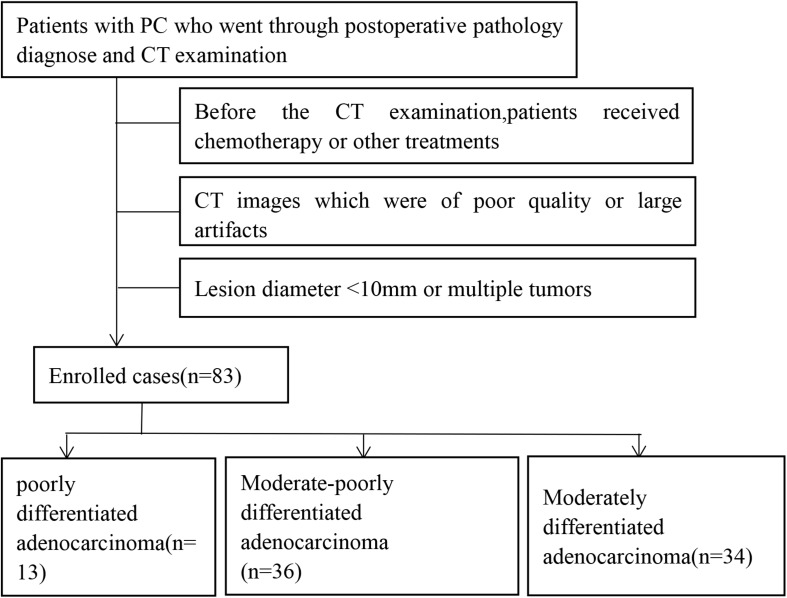
Screening and grouping flow chart of enrolled cases in this study.

A retrospective analysis was performed on patients at the Department of Imaging of the Anhui Provincial Hospital from 2013 to 2019 who met the following inclusion criteria: (1) contrast-enhanced multiphase abdominal CT scan before surgery; (2) lesion size ≥10 mm; (3) a single mass. The exclusion criteria were as follows: (1) received chemotherapy or other treatment before CT examination; (2) poor-quality images; (3) the tumor was transferred, so the patient could not undergo surgery. All included patients underwent surgical treatment within 2 weeks after enhanced CT scan. The chi-square test was used to compare the differences in the sex distribution between the groups. *P* < 0.05 was considered statistically significant. A total of 83 patients with PC diagnosed by pathology from the Anhui Provincial Hospital were collected, including 54 males and 29 females, aged from 41 to 76 years, with a mean age of 60.69 ± 9.12 years, as shown in Figure 1. The main symptoms of the patients when they came to the hospital for treatment were jaundice (74 cases, 89.1%), yellow urine (44 cases, 53.0%), abdominal discomfort (including abdominal distension or abdominal pain, 50 cases, 60.2%), itchy skin (5 cases, 6.0%), fever (2 cases, 2.4%), and diarrhea (1 case, 1.2%); in addition, one patient was found to have no obvious clinical symptoms on medical examination.

### Scanning Method

All patients fasted for 6 h before the CT scan. The examination was carried out according to the following protocol: intramuscular injection of anisodamine (654-II) 10–15 mg and 800–1000 mL of clear water orally 15 min before the scan. All examinations were performed using a multidetector CT scanner (DiscoveryHD750, Gemstone Spectral Imaging, GE Healthcare, Milwaukee, WI, United States). All patients underwent routine unenhanced/three-phase enhanced CT scans to determine the extent of the lesion. The parameters for abdominal CT were as follows: tube voltage 120 kVp, tube current 250–350 mA, slice thickness 5 mm, slice interval 5 mm, field of view 35–50 cm, matrix 512 × 512, rotation time 0.7 s and pitch 1.375. After unenhanced CT scans were obtained, each patient received 1.5 mL per kilogram of body weight of non-ionic iodinated contrast material (Iohexol, Omnipaque 300, GE Healthcare, Shanghai, China), which was administered at a rate of 3.0 mL/s using a power injector (Stellant; Medrad, Warrendale, PA, United States). The scanning delay for arterial phase (AP) imaging was determined using automated scan triggering software (SmartPrep; GE Healthcare, Milwaukee, WI, United States). AP scanning automatically began 10 s after the density in the descending aorta reached 100 HU on the monitoring scan. At delays of 30 s and 3 min after AP scanning, venous phase (VP) and delayed phase (DP) acquisitions commenced, respectively.

### Region-of-Interest (ROI) Segmentation and Radiomics Feature Extraction

The images of the arterial and VPs of all patients were collected from the CT ICPACS workstation of the Department of Radiology, Anhui Provincial Hospital, and exported in DICOM format. Two imaging physicians with 15 years of diagnostic experience in the CT diagnosis of abdominal disease used open-source software^1^ to delineate Region-of-Interests (ROIs) containing the target lesions in the arterial and venous images. Open-source ITK-SNAP software was used for ROI sketching. During the process of sketching, the operators selected the largest area of the enhancement on the AP and VP of the tumor, paying attention to avoiding the pancreatic duct, blood vessels, calcification, and necrotic cystic areas to minimize errors.

### Statistical Analysis and Clinical Predictive Model-Building

To ensure the intra- and inter-observer reproducibility, 30 patients were randomly selected and delineated by the radiologists1 for twice to calculate the intra-observer ICCs, and delineated by radiologist2 for once to calculate the inter-observer ICCs. An ICC>0.75 indicated good reproducibility. Radiologist1 finished the rest delineation.

The original images were normalized by transforming them into standard intensity ranges with a mean value of 0 and a standard deviation of 1 (z-score transformation) before the image features were extracted. AK software (GE Healthcare, Analysis Kit, Version: 3.2.0. R) was used to extract a total of 396 feature parameters, of which 42 were histogram features, nine were form factors, 154 were gray level co-occurrence matrix (GLCM) features, 11 were gray-level size zone matrix (GLSZM) features, and 180 were run length matrix (RLMs) features.

All statistical analyses were performed in R (3.5.1^2^). Patients were randomly assigned to the training and test group at a ratio of 7:3. In training group, first, Spearman correlation analysis was used to eliminate features with correlation coefficients >0.9. Next, random forest selection by filter (rfSBF) in the “random forest” package with ten-fold cross validation tests was used to select the best feature subsets in each phase. Then, the conditional inference tree “ctree” of the “train” function in the “caret” package was applied in the best sub-feature groups to train the predictive model. Afterward, the “pROC” package was applied to evaluate the discriminative performance of the model and validated in the test group. Other parameters, including accuracy, sensitivity and specificity, were calculated by the “confusion matrix.”

## Results

### Clinical Characteristics

**FIGURE 2:**
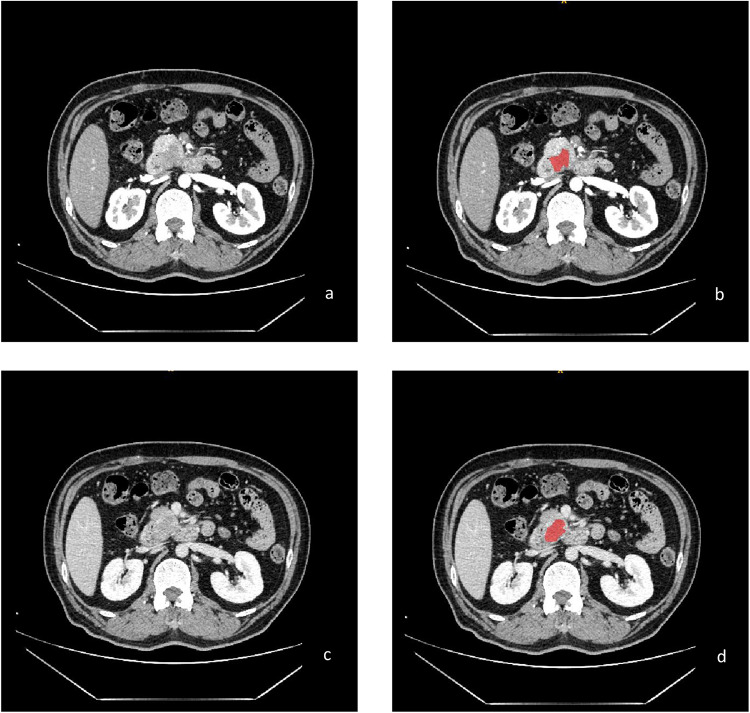
Enhanced CT images of moderately differentiated PC in the arterial phase **(a)** and venous phase **(c)**; the treatment ROI using ITK-SNAP software **(b,d)**.

A total of 83 cases of PC were included in this study. The postoperative pathological grade included 13 cases of poorly differentiated adenocarcinoma, 36 cases of moderately poorly differentiated adenocarcinoma, and 34 cases of moderately differentiated adenocarcinoma. The CT values of the AP and VP of the three groups were statistically significant (*P* < 0.05) (Table 1). Part of the images and ROIs are shown in Figure 2.

**TABLE 1:** Comparison of the clinical data of the cases grouped according to the degree of differentiation.

Degree of pathological differentiation	Number of cases (patients)	Sex		Age (year)	CT value (Hu)	
		Male	Female		Arterial phase	Venous phase
Poor differentiation	13	9	4	42∼76	52.1 ± 9.2	67.7 ± 8.4
Moderate-poor differentiation	36	24	12	43∼76	56.2 ± 9.4	72.5 ± 13.2
Moderate-poor differentiation	34	21	13	41∼75	65.7 ± 9.9	84.5 ± 11.7
*T* value		χ^2^ = 0.303		*F* = 1.213	*F* = 12.660	*F* = 13.498
*P* value		>0.5		>0.05	<0.05	<0.05

### Feature Selection and Radiomics Signature Building

**FIGURE 3:**
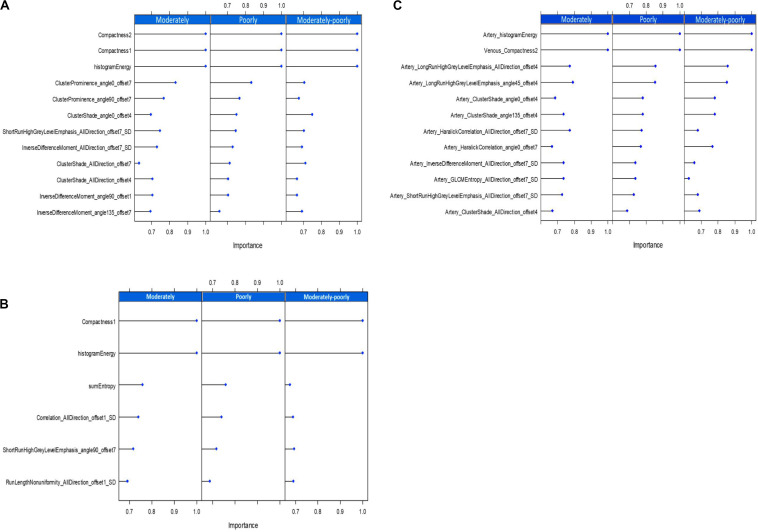
The best texture features obtained by the random forest, The arterial phase **(A)**, the venous phase **(B)**, and the combined group **(C)**.

The range of the inter-observer ICCs in the Artery phase were from 0.0096 to 1, and the intra-observer ICCs were from 0.14 to 1; In VP, inter-observer ICCs were from 0.06 to 0.94, and intra-observer ICCs were from 0.26 to 1.0, 282 features for Artery phase and 274 for VP with an ICC value bigger than 0.75 were selected for the further analysis. Spearman correlation analysis and the random forest method were used to select features. The final retained features of the arterial and VPs are shown in Figure 3. Twelve features were retained in the AP (a), six features were retained in the VP (b), and 12 features were retained in the combined group (c); the *x*-axis represents the weight of the features, the *y*-axis represents the last retained features, and the larger the weight is, the more predictive the feature is. In the AP, the three most predictive features in the poorly differentiated group, moderately differentiated group and moderate-poorly differentiated group were compactness2, compactness1 and histogramEnergy; in the VP, the two most predictive features were compactness1 and histogramEnergy; in the combined group, the two most predictive features were artery-histogramenergy and venous-compactness2. We can see that in both the AP and VP, compactness1 from “Form factor” and histogramEnergy from “histogram” show the best predictive performance. The explanation of some texture features is shown in Table 2.

**TABLE 2:** Features measured with different texture analysis methods by AK software.

Texture feature groups	Parameters
**Histogram Features** (Gray intensity information and its distribution of the lesion, for example HistogramEnergy describes the severity of the change in image brightness information, the smaller the change, the greater the Energy.)	Mean, Variance, Uniformity, Skewness, Kurtosis, Energy, Entropy
**Form Factor Features** (The shape of the lesion, For example, Compactness, describing the degree of roundness or sphericity of the lesion; if the lesion is more spherical, the Compactness value is greater.)	Volume CC, Surface, Surface Volume Ratio, Compactness, Maximum 3D Diameter
**GLCM Features** (Obtained by counting the probability of pixel pairs in different directions and step sizes)	Entropy, Inertia, Inverse Difference Moment;
**RLM Features** (obtained by counting the probability of multiple occurrences of pixels in different directions and steps)	Short Run Emphasis, Low Gray Level Run Emphasis, Short Run Low Gray Level Emphasis;
**GLSZM Features** (obtained by counting the number of pixels with the same adjacent gray value, so as to obtain the gray connected area matrix)	Small Zone Emphasis, Low Gray Level Zone Emphasis, Short Run Low Gray Level Emphasis

*GLCM, gray level co-occurrence matrix; RLM, Run Length Matrix; GLSZM, Gray Level Size Zone Matrix. All of them are used to describe the complexity of the lesion site, level changes, and the thickness of the texture and other information.*

**FIGURE 4:**
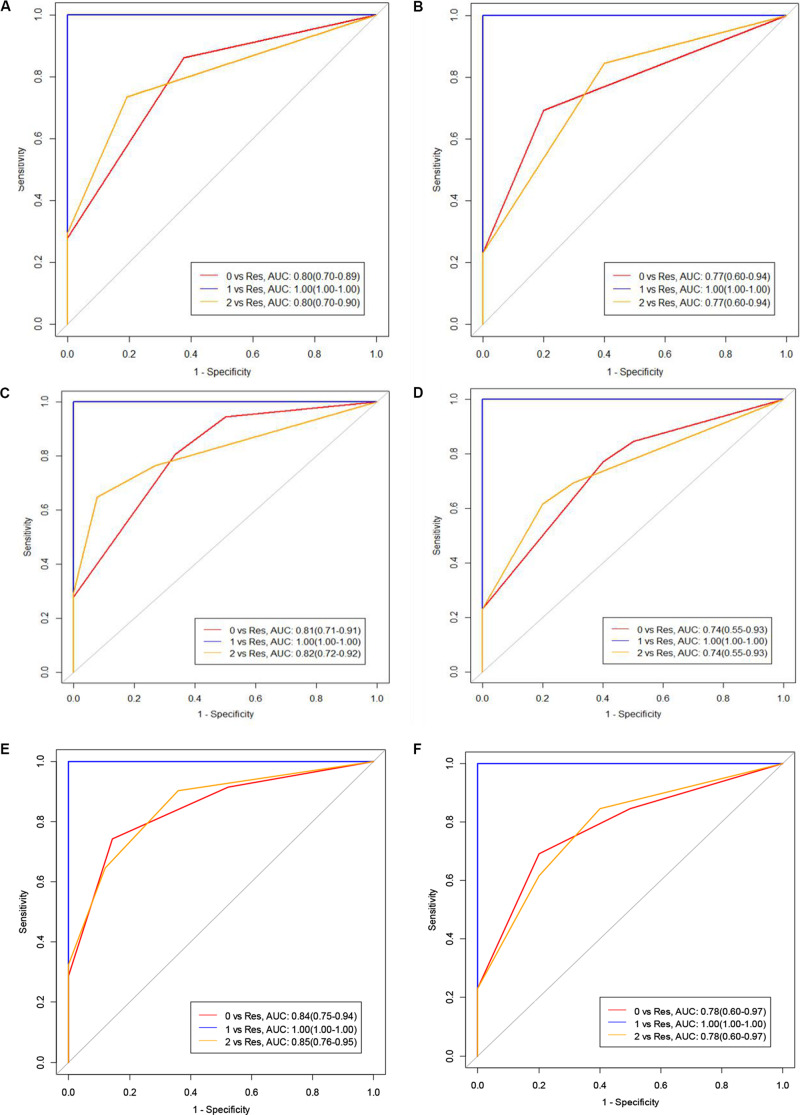
**(A,B)** represent the training and test groups with moderately differentiated, poorly differentiated, and moderate-poorly differentiated pathologies of PC. **(C,D)** represent the AUC values of the venous phase of the training and test groups with moderately differentiated, poorly differentiated and moderate-poorly differentiated PC. **(E,F)** represent the combined groups (Class 0 means moderately differentiated, Class 1 means poorly differentiated, Class 2 means moderate-poorly differentiated, Res means the rest of the cases).

The AUC(95%CI) value of the three models to differentiate the degree of differentiation in the training group and test group of PC patients were shown in Figures 4A–F. Other parameters, including sensitivity, specificity, and model accuracy were shown in Table 3, The overall accuracies in the AP and VP were 0.77(95%CI: 0.64–0.87) and 0.77(95%CI: 0.62–0.86) in the training group and 0.74(95%CI: 0.52–0.89) and 0.70(95%CI: 0.47–0.86) in the test group, respectively. We can see that neither the AUC nor sensitivity and specificity in both the AP and VP have a one hundred percent value in differentiating moderate differentiation. Additionally, the model has good performance in poor differentiation and moderate-poor differentiation, and the accuracy of the model was higher than 0.74 in both the training and test groups. Thus, it can be considered that texture analysis can be used for enhanced CT scanning and has obvious texture features. The comparison also has a certain predictive effect, and the malignancy of pancreatic cancer can be evaluated to a certain extent.

**TABLE 3:** Sensitivity and specificity of the arterial and venous phases training and test groups of PC with different degrees of differentiation.

Phase	Arterial			Phase	Venous		
	Group	Sensitivity	Specificity		Group	Sensitivity	Specificity
Training group: Accuracy 0.77(95%CI:0.64–0.87)	Class 0	0.63	0.86	Training group: Accuracy 0.77(95%CI:0.62–0.86)	Class 0	0.50	0.94
	Class 1	1.00	1.00		Class 1	1.00	1.00
	Class 2	0.81	0.74		Class 2	0.92	0.65
Test group: Accuracy 0.74(95%CI:0.52–0.89)	Class 0	0.80	0.69	Test group: Accuracy 0.70(95%CI:0.47–0.86)	Class 0	0.50	0.85
	Class 1	1.00	1.00		Class 1	1.00	1.00
	Class 2	0.60	0.84		Class 2	0.80	0.62

## Discussion

Pancreatic carcinoma is a very malignant tumor with a 5-year survival rate of <6%. And it is possible that PC will become the second leading cause of death from malignancy in the next two decades (Rahib et al., 2014; Ferlay et al., 2015). The disease is usually detected at a late clinical stage that is difficult to treat. Therefore, the preoperative evaluation of the degree of malignancy and resectability of PC is very important for the operation, postoperative treatment and prognosis of patients (Kurihara et al., 2015; Matsumoto et al., 2015). This study provides a radiomics features based machine learning model for predicting the degree of differentiation of PC before surgery. And the results show that the model has high feasibility and credibility.

Texture analysis has been shown great value in medical image preprocessing This technology is not affected by photon noise and can quantitatively measure tumor heterogeneity. It is widely used in the diagnosis, differential diagnosis and therapeutic evaluation of tumors (Davnall et al., 2012; Ng et al., 2013). Texture analysis can parameterize the potential information in the inspected image to obtain more abundant quantitative data, which will facilitate structured analysis and the processing of data. Kim et al. (2019) and Sandrasegaran et al. (2019) determined that texture analysis is useful for predicting a patient’s prognosis and resectability of the tumor, after neoadjuvant therapy for PDAC. Canellas et al. (2018) showed that texture analysis has certain feasibility in the classification of pancreatic neuroendocrine tumors. We can boldly hypothesize that this technology can also be used for pathological grading of pancreatic cancer.

Watanabe et al. (2014) showed that when more tumor-associated fibrosis is present, the stronger the tumor invasiveness, and the higher the degree of malignancy and that the overexpression of fibroblast activation protein in PC tissue causes an increase in the lesion interstitial fiber component. Blocking the contrast agent into the lesion weakens the degree of enhancement of the lesion. The results of this study are consistent with the findings of Wang et al. (2003, 2007), that the higher the malignancy of the tumor, the lower the degree of differentiation and enhancement. In addition, Eilaghi et al. (2017) showed that CT image-based texture analysis can effectively distinguish between tumor and normal pancreatic tissue, and CT texture features may become an imaging biomarker for the postoperative overall survival rate. Our study showed that the feasibility of CT image-based texture analysis for the preoperative prediction of PC differentiation.

The limitations of this study are as follows: (1) the sample size was limited, and the measured values of the parameters may be biased; (2) there was no uniform standard when selecting the ROI in this study, and manual image sketching is time-consuming and laborious; and (3) in this study, feature extraction was based on a single enhanced image for analysis. The better way is to use 3D stereo modeling to extract texture features for analysis. In actual work, imaging physicians use CT plain scans and three-phase enhancement methods to analyze and diagnose the lesions or perform multiple imaging examinations at the same time to achieve a more comprehensive diagnosis.

In summary, CT texture analysis in PC has a clear predictive value for identifying differences in tumor grading. It provides a new method for assessing the malignant degree of tumor grading. It has the advantage of being a non-traumatic examination method, it is not dependent the opinion or experience of radiologists, that still permits the accurate diagnosis of patients with cancer.

## Data Availability Statement

All datasets generated for this study are included in the article/supplementary material.

## Ethics Statement

The studies involving human participants were reviewed and approved by the Medical Research Ethics Committee of The First Affiliated Hospital of University of Science and Technology of China (Anhui Provincial Hospital). The patients/participants provided their written informed consent to participate in this study.

## Author Contributions

WW and LX contributed to conception and design of the study. ZL organized the database. GY performed the statistical analysis. ZL and GY wrote the first draft of the manuscript. ZL, WW, LX, and GY wrote sections of the manuscript. All authors contributed to the article and approved the submitted version.

## Conflict of Interest

GY was employed by the company GE Healthcare, Shanghai. The remaining authors declare that the research was conducted in the absence of any commercial or financial relationships that could be construed as a potential conflict of interest.

## Footnotes

1 www.itksnap.org

2 http://www.Rproject.org
